# Efficacy of levothyroxine on benign thyroid nodule

**Published:** 2012

**Authors:** Mohammadali Bayani, Mohammad Amani, Zoleika Moazezi

**Affiliations:** 1 Department of Internal Medicine, Ayatollah Rouhani Hospital, Babol University of Medical Sciences, Babol, Iran.; 2 Babol University of Medical Sciences, Babol, Iran.

**Keywords:** Thyroid nodules, Levothyroxine, Suppressive therapy

## Abstract

**Background:**

Suppressive therapy with levothyroxine for reducing the size of thyroid nodules has not been really accepted. The purpose of this study was to assess the effect of levothyroxine on the size of benign thyroid nodules.

**Methods:**

Forty patients with confirmed benign nodule were randomly divided into two groups. Group I received 50g/day levothyroxine for six months but group II did not take it. Sonography was used to measure the dimensions of nodules before and after six months. TSH serum levels were measured before and after treatment. This clinical trial study was registered as IRCT 201103185692 N3. The data were collected and analyzed.

**Results:**

The mean age of levothyroxine group was 41.57±9.41 and control group was 44.45±10.9 years (p=0.386). The TSH levels and longitudinal and transverse dimensions in both groups were similar (p>0.05). The TSH levels before and after treatment were 2±1.65 and 0.52±0.67 mUI/L (p=0.001). The Longitudinal and transverse dimensions before and after treatment in case group were 1.9±1.11, 1.90±1.15 and 1.44±0.90, 1.49±1.02 cm respectively (p=0.74, p=0.7, respectively) but in control group, were 2.19±1.32, 1.97±1.4 and 1.57±0.95, 1.26±0.7, respectively (p=0.35 and 0.1, respectively).

**Conclusion:**

The results show that suppressive therapy with levothyroxine lead no alteration in the size of benign nodules.

Thyroid nodule is a pathology with which endocrinologists are dealing, and is taken into consideration when it is reported by a patient or accidentally is discovered during physical examination or while performing a radiological procedure such as carotid sonography, neck CT scan and/or PET scan. The prevalence is different based on the study population, the study techniques as well as the definition used for thyroid nodules have been reported from 0.2 to 21% in various populations for palpable thyroid nodules, and is more frequent among women and older patients (1-2). Palpability means that the size of nodules should be at least 1 cm. (3). Therapeutic ways for thyroid nodules are different from nodule removal with surgery to radioactive treatment, conservative treatment with levothyroxine and mere observation. Since surgical treatment of thyroid nodules is expensive and accompanied by side effects, patients prefer observation as much as possible and medicinal treatment (4). 

However there is not yet a consensus about the safety and effect of suppressive treatment with levothyroxine to reduce the size of thyroid nodules. It has been reported that TSH leads to the control of thyroid tissue division, and T4 contributes to reducing TSH secretion through a negative feedback and, as a consequence, eventuates in decreased hypertrophy and hyperplasia in thyrocytes (5). Therefore, the purpose of treatment with levothyroxine is to inhibit TSH secretion without developing thyrotoxicosis or other unwanted symptoms. Levothyroxine should be given in an appropriate dose to maintain the serum level of TSH approximately within 0.1-0.5 mIU/l (5). 

The mentioned therapy is effective for nodules under 3 cm colloidal goiter, and colloid nodular cases (6). The aim of this study was to investigate the effect of suppressive treatment with levothyroxine on the size of thyroid nodules. 

## Methods

This randomized clinical trial study was conducted on 40 patients with single thyroid nodule who referred to the Department of Endocrinology of Babol University of Medical Sciences, patients with palpable thyroid nodules were entered to the study after being confirmed about tumor benignity based on FNAB results. To ensure the existence of single nodule, sonography was performed for all patients. The participants were 18-60 years old, the total T4 and TSH serum test results showed euthyroid status. The exclusion criteria included thyroid neoplastic lesions in patients or their family, history of neck radiation and hot nodules. None of the patients had serious cardiovascular, hepatic and renal diseases and none were pregnant. None of the participants were also under the suppressive treatment with levothyroxine or other thyroid-associated drugs prior to the study. All subjects were informed about the study and informed consent were obtained by all the patients. The patients were randomly divided into two groups; group I (n=20) received levothyroxine with the initial dose of 50 μg/day (produced by Iran Hormone Company), in which levothyroxine dose was coordinated with TSH serum level after 6 months suppressive treatment in order to bring TSH level to less than 0.5mu/1. The second group (n=20) received no medication, and both groups were followed up for 6 months. The serum levels of TSH was assessed before and after the study using commercial kits with normal levels of 0.5-4.5 mUI/l. Thyroid sonography was performed by one physician prior to the study and 6 months after suppressive treatment, and for the decreased longitudinal and transverse dimensions reported in sonography, responses to suppressive therapy was defined as complete response (>50% reduction in longitudinal and transverse dimensions of nodules), partial response (20-50% reduction in the longitudinal and transverse dimensions of nodules), no response or increased in size. 

**Statistical analysis: **The data were analyzed using SPSS version 18. The data presented as mean ± standard deviation. t- test was used for the comparison of quantitative variables in each group and paired test for the comparison of quantitative variables between the two groups, and p<0.05 was considered meaningful. 

## Results

Forty patients (34 females and 6 males) with the mean age of 43.05±10.17 years participated in the study. Group I received levothyroxine with the initial dose of 50 μg/day, and the other 20 patients in the control group received no medication. Both groups were evaluated again after 6 months. The baseline data of both groups are shown in table 1. No significant differences were found in terms of age, gender, TSH levels and longitudinal and transverse dimensions of the nodules between the two groups. 

The mean level of TSH was 1.58±1.06 mUI/L before the therapy, and it reduced to 0.52±0.67 mUI/L six months after levothyroxine treatment in this group (including 17 females and 3 males), demonstrating inhibition of TSH secretion (p=0.001). In control grou,p the mean level of TSH was 1.6±1.18 mUI/L before the therapy and was 1.32±0.98 six months later without any significant change (p=0.442). There was also no significant difference between the primary and the secondary dimensions of thyroid nodules in both groups (table 2). Likewise, the nodule size did not display any remarkable differences compared to primary dimensions in the control group six months after the study (table 2).

At the end of the study, the subjects were investigated in three groups based on treatment responses regarding the longitudinal and transverse dimensions of thyroid nodules:

The complete response was seen in 2 cases of control group, partial response was seen in seven cases of levothyroxine, and 8 cases of control group. No response or increased size was seen in 13 cases of levothyroxine and 10 cases of control group.

**Table 1:** The base clinical and laboratory data of two groups (n=20)

**pvalue**	**Control Mean±SD**	**Levothyroxine ** **Mean±SD**	** Group ** **Variable**
0.386	44.45±10.9	41.57±9.41	Age (yr)
0.390	1.65±1.18	2.00±1.65	TSH
0.523	2.19±1.32	1.94±1.14	longitudinal dimensions (cm)
0.662	1.57±0.95	1.44±0.90	Transverse dimensions (cm)

**Table 2:** Compare primary dimensions of nodules and 6 months after treatment

**pvalue**	**Secondary**	**Primary**	**Dimensions** ** (cm** **)**	**Groups**
0.735 0.657	1.90±1.15 1.49±1.02	1.9±1.11 1.44±0.90	longitudinal Transverse	Levothyroxine (n=20)
0.354 0.109	1.97±1.40 1.26±0.70	2.19±1.32 1.57±0.95	longitudinal Transverse	Control (n=20)

## Discussion

In this study, six months treatment with levothyroxine in patients with benign thyroid nodules led to no change in nodules size compared to the control (without placebo), and only 2 out of 20 cases showed 50 percent reduction in nodules size in the control group. 

There are some contradictions about the effect of levothyroxine on reducing the size of thyroid nodules (7). The main purpose of treatment with levothyroxine in patients with thyroid nodule is to decrease TSH secretion, since it is believed that TSH is a growth factor for thyroid tissue (8), and it has also been shown that TSH increases growth factor binding to the thyroid follicles in cell culture media (9). In addition, thyroxine itself can cause inhibited growth of thyroid tissue (10). In the present study, TSH was successfully located within the range of 0.52±0.67 mUI/l after six months treatment with levothyroxine. 

Papini et al. showed that 12 months suppressive therapy with levothyroxine on 51 patients with thyroid nodules contributed to size reduction in 45% of patients compared to 26% in the control group. The mean TSH reached to 0.06±0.06 mUI/l in drug-treated group (11). In another study by Lima et al. in 1997 on 54 patients with one-year levothyroxine treatment, it was found that none of the patients had significant reduction in nodule size. Suppressive therapy in this study brought the mean TSH level to less than 0.1mUI/l in drug-treated group (12). Zelmanovitz et al assessed 45 patients with thyroid nodules and concluded, that after one year of follow-up, with suppressive treatment with levothyroxine reduction in nodule size was seen in 17% of patients, and there was also prevention of nodule growth in 10% of cases (13). Larijani et al. examined 62 patients in a double blind clinical trial in 1999, and observed that, after 24 months follow-up, 6 subjects in levothyroxine and 4 cases in placebo groups had more than 50 percent reduction in nodule size. The mean level of TSH was 0.17±0.2 mUI/l in this study (14). In 2002, in a randomized double blind study by Wemeau et al. in 123 patients with thyroid nodules, more than 50 percent decrease in nodule volume was shown after 18 months follow up in 26% and 16% in levothyroxine and placebo groups, respectively. However, the TSH level reached to 0.73±0.8 mUI / l in levothyroxine group (15). In 2006, TSAI et al. exhibited that from 30 patients receiving levothyroxine for 6 months, 11 cases were reported to have more than 50% reduction in their thyroid nodule volume after reaching the mean TSH to 0.08±0.02 mUI/l. In this study, the patients with higher levels of serum thyroglobulin did not show better response to suppressive treatment (5). 

The present study demonstrates that suppressive therapy with levothyroxine leads no alteration in the size of benign thyroid nodules. 
